# Barriers and Facilitators in Adolescent Psychotherapy Initiated by Adults—Experiences That Differentiate Adolescents’ Trajectories Through Mental Health Care

**DOI:** 10.3389/fpsyg.2021.633663

**Published:** 2021-03-05

**Authors:** Signe Hjelen Stige, Tonje Barca, Kristina Osland Lavik, Christian Moltu

**Affiliations:** ^1^Department of Clinical Psychology, University of Bergen, Bergen, Norway; ^2^Finnmark Hospital Trust, Hammerfest, Norway; ^3^District General Hospital of Førde, Førde Hospital Trust, Førde, Norway

**Keywords:** adolescent, mental health, helpful, unhelpful, therapy, dropout, treatment, qualitative study

## Abstract

Mental health problems start early in life. However, the majority of adolescents fulfilling the criteria for mental health disorders do not receive treatment, and half of those who do get treatment drop out. This begs the question of what differentiates helpful from unhelpful treatment processes from the perspective of young clients. In this study, we interviewed 12 young people who entered mental health care reluctantly at the initiative of others before the age of 18. Their journeys through mental health care varied significantly despite sharing the same starting point. Our analyses resulted in a model of three trajectories. We describe relational and structural facilitators and obstacles within each trajectory and have formulated narratives highlighting core experiences differentiating them. Trajectory 1 (*I never saw the point – Being met as a case*) was characterized by a rapid loss of hope, leading the adolescents to conclude that mental health care was not worth the investment. Trajectory 2 (*I gave it a go, but nothing came of it – Being met by a therapist representing a rigid and unhelpful system*) was characterized by a lingering hope that never materialized into a constructive therapeutic process despite prevailing efforts by both therapists and adolescents. Trajectory 3 (*Something good came of it – Being met by a therapist who cares and wants to help*) was characterized by genuine meetings, allowing the therapist to transform from an unsafe stranger into a safe, competent, and benevolent adult. We discuss how our results have implications for understanding agency displayed by adolescent clients in therapy, therapist flexibility and authenticity, service organization, and attributional processes influencing clinical judgment and therapeutic processes when adolescent psychotherapy has a difficult starting point (i.e., initiated by adults).

## Introduction

Research indicates that only about 25% of adolescents with mental health problems have been in touch with mental health care the past year (Gulliver et al., 2010). Many young clients come to therapy at the initiative of others (de Haan et al., 2013), meeting mental health care at a less-than-optimal starting point considering the importance for adolescents to assert agency in therapy (Gibson and Cartwright, 2013). Moreover, 28–75% of young clients quit treatment prematurely (Swift and Greenberg, 2012). Their reasons are diverse: Some are dissatisfied, some perceive (whether or not the therapist concurs) that they have achieved what they wanted, and some quit because of difficulties outside therapy (O’Keeffe et al., 2019). In-session events and therapist behaviors are linked to adolescents dropping out of treatment (O’Keeffe et al., 2020), but it has been difficult to predict which young people are at risk of dropping out (de Haan et al., 2013; O’Keeffe et al., 2018). Taken together, research indicates that the existing clinical practice in mental health care does not succeed in providing treatment that is perceived as accessible or helpful for many adolescents. Therefore, to improve services, it is important to understand what differentiates helpful from unhelpful treatment from the perspective of adolescents, especially when therapy has a difficult starting point, e.g., when adolescents enter mental health care at the initiative of others.

General psychotherapy research has shown that the client–therapist relationship affects outcomes in both adults (Horvath et al., 2011) and adolescents (Shirk et al., 2011). Research explicitly exploring the client–therapist relationship in therapy with young people has reported their distinctive notions of the relationship: Relative to adult clients, adolescent clients expect it to be less formal, less hierarchical, and more like a friendship (Everall and Paulson, 2002; Gibson et al., 2016; Løvgren et al., 2019). Adolescents more readily form an alliance when they perceive the therapist as genuine, accepting, respectful, interested, supportive, and trustworthy (Everall and Paulson, 2002; Binder et al., 2011; Sagen et al., 2013; Gibson et al., 2016; Lavik et al., 2018; Løvgren et al., 2019). By contrast, the adolescent client naturally finds it unhelpful to feel misunderstood or unappreciated, and professionalism may be perceived negatively as distance-inducing (Levitt et al., 2016). Given that autonomy and agency are already key developmental tasks in adolescence, it is important to adolescents that they assert agency in therapy (Gibson and Cartwright, 2013); that many of them have come to therapy at the initiative of others places a particular burden on the therapeutic relationship (de Haan et al., 2013). Perhaps surprisingly, though, most research on psychotherapy with adolescents does not specify who initiated the therapy. Very little is, therefore, known about how adolescents experience coming to therapy at others’ initiative. Moreover, therapists often perceive adolescents as a difficult group to engage in therapy (Everall and Paulson, 2002), and research on adult psychotherapy has found that therapists’ attitudes toward their clients form quickly and influence clinical judgment, including prognosis and diagnostic assessment (Strupp, 1993). Therapy is, therefore, constituted by unique encounters between two persons, in which both parties bring with them experiences and expectations influencing the evolving interaction and relationship (Bucci et al., 2016; Råbu and Moltu, 2020).

A few studies have illuminated how young people manage autonomy within the therapeutic relationship (Gibson and Cartwright, 2013; Løvgren et al., 2019). Løvgren et al. (2019) found, for example, that adolescent clients manage a sense of agency by carefully controlling what they say to the therapist and when they say it. Considering the issue of agency in light of how adolescents conceive of a helpful client–therapist relationship as like a friendship, we can begin to understand why the first meeting in adolescent therapy is so important. Research shows, for example, that client–therapist agreement on a strong alliance in the first session is associated with an eightfold increase in the odds of a favorable outcome compared with dyads in which therapist and client both assess the alliance as poor (van Benthem et al., 2020). Some studies also indicate that a strong therapeutic alliance might be particularly important for a good outcome when the young person has a history of poor attachment experiences (Zack et al., 2015).

There is a need, then, to better understand adolescent psychotherapy processes when therapy has a difficult starting point so that adolescents with mental health problems will be willing to persevere with therapy long enough to reap the benefits from various efficacious treatment approaches that have been developed. In this article, we, therefore, explore how adolescents coming to therapy at the initiative of adults experience their journeys through mental health care and what, from their perspective, differentiates helpful from unhelpful experiences with therapy.

## Materials and Methods

### Study Setting

The study was conducted in the context of a welfare system, in which children and adolescents receive free medical, dental, and mental health care until they are 18 years old. The idea for the current study was conceived while the first author was working as a psychologist in an outpatient clinic for children and adolescents. Meeting adolescents weekly that were referred because the school put pressure on the parents to seek help on behalf of the adolescent or because child protective services thought the adolescent needed help or because the parents pushed them to go, she wanted to learn more about how adolescents experienced coming to mental health care at others’ initiative, how therapy could become helpful with that starting point, and how therapists understood and related to this phenomenon. Some years later, when working as an associate professor at the university, she, therefore, initiated a multisite qualitative study involving practitioner-researchers and colleagues at the university to explore this phenomenon from the perspectives of the therapists and the adolescents.

### Design

To explore how adolescents experience therapy when the starting point for therapy is difficult, we chose to recruit adolescents who had experienced adult-initiated referrals to mental health care. Given this starting point, we expected challenges related to recruitment as the inclusion criteria meant adolescents had to trust that we, as adult researchers, were interested in their experiences and perspectives despite their previous experiences with adults overriding their perspective and pressuring them into contact with mental health care services. We, therefore, put a lot of thought into study design and cooperated closely with a youth user organization (Forandringsfabrikken) in all phases of planning and designing the study, including recruitment strategies and materials and the formulation of interview questions.

To access the adolescents’ perspective, we chose a design whereby participants were invited to two individual semistructured interviews with the same researcher. This would enable the participants to get to know the interviewer slightly, allowing them to provide the information they were comfortable with in the first interview while also knowing they had a second opportunity to share and expand on their perspective. We also wanted to provide the adolescents control over the information they shared and enable them to utilize the ways of sharing with which they were most comfortable. We recognize that interview as a format keeps so much control in the hands of the interviewer and is on the adult’s premise. As the interview occurs at one specific point in time, the data generated consist of the experiences available to the interviewee at that time. Although we invited participants to two interviews, we wanted to include more flexibility in the design. The adolescents, therefore, had the opportunity to write down or record relevant experiences, reflections, or thoughts they wanted us to include in the data material. Each participant received a pin code–protected digital recorder at the first interview in addition to instructions on how they could password-protect word documents to ensure confidentiality.

### Recruitment

We used several strategies to distribute information about the project and getting in touch with adolescents who had relevant experiences. Posters were placed in the waiting areas of the eight clinics included in the study along with business card–size information cards. The project’s title and research question (What is it like to come to a mental health care setting at others’ initiative?), inclusion criteria (12–18 years of age and enrollment in mental health care at others’ initiative), and the project’s webpage and contact information were listed on the posters and cards. Effort was devoted to creating an engaging and interactive webpage inviting adolescents to read about the project and what the participation entailed, and it included pictures and information about the researchers, the potential benefits and disadvantages of participation, and a messaging service for potential participants to contact the project leader (first author). Recruitment was extremely slow despite efforts to tailor the materials and recruitment strategies to the target group. Within the first 6 months, only one participant had been recruited. Hence, we expanded the inclusion criteria to include youths who were >18 years at the time of recruitment, but who had received mental health care at the initiative of others before the age of 18. The user organization then distributed information about the project to their members, resulting in 10 participants volunteering to participate. An additional participant was recruited through the project’s webpage.

### Participants

A total of 12 participants (11 females) volunteered to participate, and all were included in the project. Ten participants were involved in the user organization Forandringsfabrikken, and two participants volunteered through the project’s webpage. The participants’ ages ranged from 15 to 19 years at the time of the interview (mean age 17) and ranged from 6 to 15 years at the time of their first contact with mental health care. Child protective services had initiated treatment contact for five participants. For the remaining participants, treatment had been initiated most often as a joint effort between parents and teachers or school nurses or general practitioners. All participants had received treatment after the age of 14 and had at least one treatment period that extended over a prolonged period (>3 months). All participants had received individual treatment with the involvement of parents and family. The specific therapeutic approach provided differed between participants, reflecting the breadth of evidence-based treatment approaches provided by therapists in the public mental health services for children and adolescents. Most participants received treatment for more than a year, and five participants received more than one period of treatment. Child protective services were involved in the lives of 7 of the 12 participants because of an unsatisfactory care situation or the severity of symptoms exhibited by the adolescent. Hence, half of the participants had severe negative relational experiences prior to entering treatment.

### Data Collection and Data Material

All participants signed an informed consent form prior to the interviews. In cases in which the participant was below the age of consent (16 years), parents and adolescents signed separate consent forms. Participants were interviewed from May 2017 through December 2018 by one of four researchers (first, second, and last authors plus an additional interviewer). Interviews lasted from 45 to 150 min with most interviews lasting about 90 min. The interviewers, who were all clinical psychologists and researchers, focused on creating a safe environment by providing the adolescents with opportunities to share their perspectives of entering mental health care at others’ initiative using open-ended questions, active listening, probing, summaries, and other facilitative techniques. Although we prioritized following the initiative of the adolescents and the experiences they shared, the prepared questions in the first interview centered around the experience of entering mental health care based on others’ initiatives, and the focus during the second interview centered on the experience of undergoing treatment (see Table 1).

**TABLE 1:** Interview guide for the two interviews with the adolescents, developed in cooperation with young people in the user organization Forandringsfabrikken.

Interview 1	Interview 2
Can you tell me how you experienced coming to CAMHS? What happened? *How would you like to be met?*	Can you first tell me what treatment you are receiving now? *If you could choose, would you continue treatment?*

How should the adults at CAMHS be? What should they do? *How should an adult be so you can feel safe and tell the truth?*	How do you experience the treatment you are receiving now? *Who decides what you focus on?* *What is helpful with the treatment you are receiving now?* *What does your therapist do that is important for you, the way things are now?* *What is less useful/what could make the treatment more helpful?*

How did you end up at CAMHS? What happened? *Whose idea was it?* *What did you get to know before you got to CAMHS?* *Did you feel you had any choice coming to the CAMHS?*	How have you experienced receiving treatment at CAMHS thus far? *Is it different attending CAMHS now compared to the beginning?*

What did you expect/imagine when you got to know that you were going to the CAMHS? *What were you afraid would happen?* *What did you hope would happen?* *What did you know about the CAMHS already?*	What could have been done differently if CAMHS should become more helpful/provide better help? *Can you give me three pieces of advice for how CAMHS can become as helpful as possible for children and adolescents in the future?*

*The interviews were semistructured, following the adolescent’s lead. possible follow-up questions in italic.*

Researchers wrote down impressions from the interviews and field notes shortly after completing each interview. This information was shared between the researchers to allow for adjusting the focus in the interviews based on the emerging data and experiences using the interview guides. Following the first interview, the interview guide was adjusted slightly. Although we invited all participants to participate in two interviews, some decided before the first interview that they only wanted to participate in one interview. Others did not want to participate in a second interview when they were contacted following the first interview. Thus, the data consists of 18 interviews from 12 participants. All interviews were audio-recorded and transcribed verbatim. None of the participants used the opportunity to provide written or recorded material in addition to the interview data.

### Data Analysis and Reflexive Processes

Reflexive thematic analysis, often conceptualized as involving six phases and emphasizing the researchers’ role in knowledge production (Braun and Clarke, 2006, 2019), was used as a general framework to guide the data analysis. In the following, each phase of the analytic process is detailed, including reflexive processes, to ensure transparency that allows the reader to assess trustworthiness and transferability of the findings (Morrow, 2005; Stige et al., 2009). The analysis of the data alternated between bottom-up and top-down processes but was always guided by the dedication to relating to and understanding the participants’ experiences from their perspective. The research process was firmly planted in the phenomenological tradition of being attuned to experience (Van Manen, 2014). At the same time, we acknowledge that we, as meaning-making beings, always influence the way we understand and interpret the world and, therefore, need to pay attention to the way our positions in the world influence the research process. Thus, we are situated in a hermeneutic tradition with our focus on reflexivity (Alvesson and Sköldberg, 2009; Stige et al., 2009). Hence, a team-based hermeneutical–phenomenological approach was used throughout the research process (Laverty, 2003; Binder et al., 2012).

To start the analytical process, all authors read the transcripts thoroughly, taking individual notes of what stood out as significant in the material and what caught their interest. This phase was explorative and inductively driven with a dedication to tune into the lived experiences shared by the adolescents in the interviews. Following this initial reading of the material, the four authors met for 1 day, which served both reflexive and analytic functions. This meeting took advantage of the outsider position of the third author, who had not been part of the design or data collection. All the authors shared their starting point (why they became interested in the project), their experiences interviewing participants and reading the data, their reflections on how their background and interests influenced their reading of the material, and their suggestions for analytical foci.

All authors are clinical psychologists, have experience working clinically with adolescents, and share a strong commitment to offering treatment that is experienced as meaningful for the adolescents we meet. We were deeply touched by the participants’ shared experiences with their complex layers; the courage and perseverance they had shown; and the discomfort, betrayals, and pain they had endured. As therapists, we also felt shame relating to some of the situations participants shared, and we were genuinely surprised that so many of them had experienced the treatment contact as somewhat helpful despite not wanting treatment initially. We had lengthy discussions about which analytic focus we should pursue, balancing between the wish to do justice to the complexity of the adolescents’ experiences, contributing to the field, having space to present the findings properly within the word limits of an article, and avoiding the salami-slicing problem (i.e., dividing the data material on as many articles as possible).

Through reading and discussing the data, we established a preliminary structure of the highest level of abstraction, consisting of three different trajectories through mental health care. In the next phase, we explored these preliminary trajectories in depth, trying to untangle what differentiated the experiences of the adolescents within the different trajectories. To this end, the first, second, and third authors individually read through all interviews to record which trajectory was described in each interview. We then conferred and reached consensus on the placement of each participant’s journey through treatment in terms of the three trajectories. We found interesting discontinuities, as the trajectories of five participants had changed during their treatment experiences. Thus, the first author read through the transcripts of the eight interviews with these five participants again, identifying and placing all sections in the interviews in their respective trajectories. This second phase of analysis was an empirically informed top-down process in that we chose to sort the data by trajectory and to have an analytical focus on the relational and structural facilitators and obstacles experienced within each trajectory. It was, however, a simultaneous inductively driven process as we sought to stay as close to the participants’ language and experiences as possible when coding the data.

The first and second authors then did a line-by-line coding of the transcripts within each trajectory, marking and naming all parts of the text relevant to the preestablished categories of structural and relational facilitators and obstacles. Following this initial thematic analysis within the trajectories, the first and second authors met to deepen their analysis of the relational and structural facilitators and obstacles within each trajectory. We then compared nodes and meaning patterns across trajectories, attempting to identify core experiences influencing which direction the participants’ journeys took through treatment. The results of this analysis were formulated through three narratives.

We convened a new meeting for reflexive and analytical purposes after the results were sent to the third and fourth authors. They, in turn, presented their reflections, experiences of resonance of the presented analysis with their initial reading of the text and their understanding of the participants’ experiences. The first and second authors shared their perspectives on the process thus far, including how their preunderstanding and engagement in the phenomena were influencing the process (e.g., the first author’s fear of presenting the results in ways that could be understood as blaming the adolescents for their bad experiences in treatment). The first author used the input from the meeting to deepen the analysis further. Then, the analyses were sent to the second author and then to the third and fourth authors, and consensus on the thematic structure of each trajectory and core experiences identifying each one was reached through discussions and correspondence between the authors.

### Ethics

As described above, a high degree of ethical reflection was present throughout the project as we were approaching a vulnerable group of participants who had experienced the overriding of their perspective on the need for mental health care by adults. We, therefore, took several measures to ensure the adolescents’ participation was voluntary: their informed consent was given, they experienced being met with respect and dignity, and they had opportunities to share their experiences with mental health care on their own terms. The project followed the ethical principles stated in the Declaration of Helsinki (WMA, 2013), and it was approved by the regional committee on medical and health research (2016/1384/REK Vest).

## Findings

**FIGURE 1:**
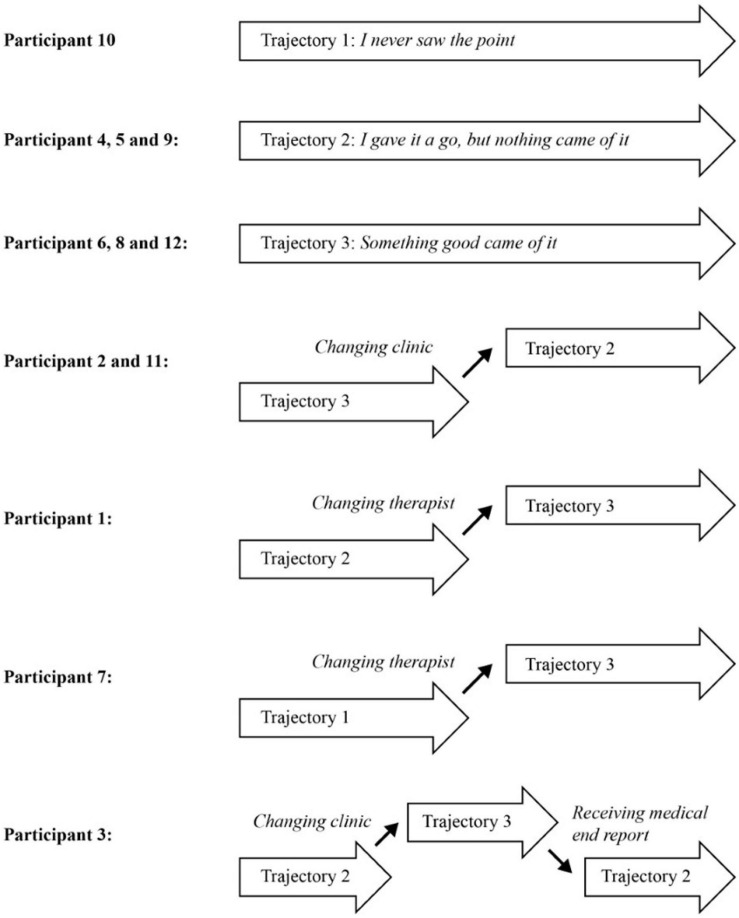
Overview of the twelve participants’ trajectories through mental health care at others’ initiative.

During the analysis of the participants’ experiences with mental health care, it became clear that some participants never really tried to engage in a therapeutic project, nor did they feel they benefitted from their treatment (Trajectory 1). Some participants had tried to benefit from it without succeeding (Trajectory 2), and others had given treatment a chance and experienced a reward for their efforts (Trajectory 3). Importantly, all participants talked about both good and bad experiences during their treatment and how they were active in navigating and making choices within this experienced field of opportunities. When analyzing the data for the three trajectories, we found that one participant followed Trajectory 1 (*I never saw the point – Being met as a case*) throughout treatment, and one other participant had experiences from this trajectory. Three participants had followed Trajectory 2 (*I gave it a go, but nothing came of it – Being met by a therapist representing a rigid and unhelpful system*) throughout their treatment, and four other participants had some experiences from this trajectory. Three participants had followed Trajectory 3 (*Something good came of it – Being met by a therapist who cares and wants to help*) throughout their treatment with five additional participants having some treatment experiences falling within this trajectory. An overview of the 12 participants’ journeys through mental health care can be found in Figure 1. As illustrated in the figure, five participants experienced switching between trajectories during their contact with mental health care. The analysis of to what participants attributed the switches pointed to changes in clinic or changing therapists within the same clinic. For one participant, receipt of the medical end report changed the entire treatment experience for her as she realized that her therapist had not been transparent during their treatment contacts and had diagnosed her without her knowledge.

In the thematic analysis of relational and structural facilitators and obstacles within each trajectory, we developed a detailed and nuanced picture of how participants within each trajectory experienced their treatment contacts (see Table 2 for theme formulations and descriptors). It is evident in Table 2 that there was a large degree of agreement across participants and trajectories regarding what constituted relational and structural facilitators and obstacles and that the trajectory an individual followed depended on how the balance between different facilitators and obstacles were experienced and handled in the clinical encounters. The therapist being authentic, showing who he or she was as a person, and being interested in who the adolescent was as a person were experienced as a potent relational facilitator across trajectories. Similarly, the adolescents’ preconceptions of mental health care services and therapists, whether it was based on friends’ bad experiences or information on the Internet, functioned as a structural obstacle across trajectories.

**TABLE 2:** Overview of theme formulations (in italic) and descriptors within each category for the three trajectories.

		Relational facilitators	Relational obstacles	Structural facilitators	Structural obstacles
**Trajectory 1**: *I never saw the point – Being met as a case* (2 participants)	Theme formulation:	*Getting a sense of the therapist as a person*	*I am a person, not a case to solve*	*The treatment has potential*	*This thing is not for me*

	Descriptors:	-Authenticity/Genuineness	-Disinterest -Insensitivity -Rigidity -Negative therapist preconceptions -Narrow focus on problems -Therapist insecurity -Therapist inauthenticity -Feeling unwanted -Adolescent not open for help	-Flexibility -Predictability	-Lack of flexibility -Predefined client role -Involvement of parents in treatment -Narrow focus on assessment and diagnosis -Forced to attend treatment -Preconceptions of mental health care and therapists

**Trajectory 2**: *I gave it a go, but nothing came of it – Being met by a therapist representing a rigid and unhelpful system* (7 participants)	Theme formulation:	*Coming to the foreground as persons*	*Meeting a wall of professionalism*	*This treatment can work for me*	*Who is this thing made for?*

	Descriptors:	-Ok first impression -Therapist continuity	-Disinterest/not using time to get to know the adolescent -Inattentiveness -Sense of rush/Focus on efficiency -Misunderstandings -Lack of transparency -Disrespect -Violation of trust -No chemistry/mismatch -Unbalanced focus on problems -Rigidity -Poor communication skills -Therapist insecurity -Adolescent not open for help	-Flexibility -Significant other has faith in the benefit of treatment -Former positive experience with treatment -Diagnosis offer explanations	-Preconceptions of mental health care and therapists -Unpredictable information flow -Lack of flexibility -Notetaking during sessions -Case notes create insecurity -Therapist turnover -Narrow focus on assessment and diagnosis -Treatment rooms impersonal -Forced to attend treatment

**Trajectory 3**: *Something good came of it – Being met by a therapist who cares and wants to help* (8 participants)	Theme formulation:	*Coming to the foreground as persons rather than roles*	*Not finding the rhythm together*	*This treatment is made for me*	*What is this thing?*

	Descriptors:	-Engagement/Interest -Competency -Sensitivity -Accept/Openness -Flexibility -Authenticity/Genuineness -Benevolence -Transparency -Trustworthiness	-Rigidity -Inattentiveness -Instability/unpredictability -Violation of trust -Time pressure/Sense of rush -Use of irony -Narrow focus on problems -No chemistry/Mismatch -Misunderstandings	-Flexibility -Continuity -Time -Predictability -Focus on confidentiality -Information -Support to stay in treatment -Improvement/Getting techniques that work -Pragmatic use of diagnosis -Safe treatment rooms	-Preconceptions of mental health care and therapists -Inconsistent focus in sessions -Narrow focus on diagnosis -Notetaking during sessions -Discontinuation of treatment during holidays and illness -Information material unfitting -Waiting room unsafe -Treatment rooms small and unsafe -Difficult changing therapist -Involvement of parents in treatment

Large differences in the amount of structural and relational facilitators and obstacles experienced within each of the three trajectories influenced participants’ access to hope and the perceived room for opportunities to be navigated. For example, participants in Trajectory 3 experienced an overweight of relational and structural facilitators and found ways to utilize these resources to their advantage despite also facing numerous relational and structural obstacles. In contrast, participants in Trajectories 1 and 2 experienced few relational and structural facilitators but an abundance of relational and structural obstacles. Despite this situation, participants in Trajectory 2 continued to try, hoping they would benefit from treatment, while participants in Trajectory 1 quickly decided that mental health care was not worth their investment.

What then, were the core process and experiences defining each trajectory, and where did they part ways? In the following section, we present three narratives representing and illustrating the thematic analysis presented in Table 2. The narratives show and clarify our interpretation and assessment of the core processes and experiences influencing the trajectories the participants took through mental health care given that all of them came to mental health care at the initiative of others. In the quotes, the abbreviation of child- and adolescent mental health services (CAMHS) is used as a generic term to translate the participants’ reference to the national mental health services for children and adolescents.

### Trajectory 1: I Never Saw the Point — Being Met as a Case

The two participants in Trajectory 1 came reluctantly to treatment but were willing to give it a chance. The decisive experiences that seemed to nudge them into Trajectory 1 were not related to their starting points but rather the interplay between their starting point and the ways their therapists met them, resulting in the profound feeling that there was no point in even trying to get anything out of treatment.

The participants in Trajectory 1 felt that the mental health care system, with its expectations and procedures, was scary and unsafe. The participants experienced their therapists’ inability to detect these concerns, resulting in a complete failure to make adjustment to reduce the strain on the adolescents:

Well, I didn’t really know why I was sent to the CAMHS, it was just my mum who made arrangements with school and stuff. So…and then that woman [therapist] just carried on and sort of asked lots of questions, and I felt sort of like she took it for granted that I would just sort of unfold my whole life and everything I felt inside to her while my mum, like, a person I don’t trust at all, was there. Erm, then, and then I felt that it was a bit lame that she didn’t like, she didn’t even ask me if it was OK that I had my mum there. (Participant 7).

The two participants in Trajectory 1 experienced a rapid loss of hope in the mental health care system’s ability and interest in helping them. Participant 7, for example, decided she wanted to get out of mental health care as quickly as possible following the first session, at which she experienced that the therapist violated her integrity by involving her mother without being sensitive to the impact she had on her:

Interviewer: How, when you and your mother left the place after that first appointment and talked about it, how, what was it like for you then, how did you do inside?

Participant: Erm… I had firmly decided that I would not be there for very much longer at least at the CAMHS. And I didn’t think, I didn’t need that, and they couldn’t help me anyway, so. Like, I didn’t believe that they could help me, that they could do any good, almost like. (Participant 7).

Participant 10 also experienced a rapid downward spiral, depriving her of all hope of receiving any help from the mental health care system. Child protective services had pressured her to enter treatment but gave her time to do so; thus, she was willing to try the treatment when she entered mental health care:

First I got… erm, yes, first they [child protection service] asked me if I wanted to go there, so it was completely up to me, so I said no, I don’t want to. I have thought about it, I don’t need that. And then a few months passed, and then yes, I thought it wouldn’t do any harm to try, I didn’t know very much about the CAMHS then. Back then, I thought they could tell my dreams and what they meant and stuff […] So had thought that yes, maybe they can help. And then I started there, with like single, like totally common, yes, and it got off to a bad start, so yes. (Participant 10).

Her experience of entering therapy was characterized by a total lack of interest in her as a person. She felt she was a puzzle they had to solve, not a person they wanted to help:

Yes, they [the therapists] sit with, with their legs crossed as if, or together… yes they sit with notes, notepads, as if it’s their job, there is no…they should at least pretend as if it isn’t just their job. Because they are sat there with a person, and then it’s, we’re not a thing exactly. We are not a case, we are a person they are meant to help. Not a case to solve. […] I was a referral for them to solve, not a life for them to fix. (Participant 10).

She subsequently felt the therapists had already made up their minds about who she was, what she struggled with, and what she needed based on the information in the referral and their preconceptions of adolescents referred for mental health care. She experienced that they showed no interest in checking out whether these preconceptions were accurate or not. The therapists’ access to second-hand information also made the conversations unpredictable, thus unsafe, and it hindered their curiosity about and sensitivity to her:

Yes, so they [the therapists] knew a lot of stuff about me that I didn’t know that they knew, so suddenly they bring up, yes what was it like when blah blah blah happened, and it was like… OK. So it was… the thing about a fresh page, it… […] Yes, they could have gotten to know me themselves, like, instead of just getting a whole, what, sheet [referral]. […] She wasn’t interested in what I was saying, she just, well, did her job. (Participant 10).

In summary, the treatment contacts rapidly induced or increased insecurity and decreased hope:

It was very much like, instead of going somewhere to talk, it was a place where you are sick in your head, go there to get treatment, like. And that was a scary thought (laughs). Because I lived a completely normal life, and I am surrounded by friends, and yeah. So, it started making me, what’s it called… become more insecure in myself, instead of them helping me, I began having doubts, I thought, do others see me this way, does everyone see me this way? And then I became, like, what’s it called, not self-esteem but self-image. (Participant 10).

Both participants’ regarded treatment as an exercise or duty and did not try to engage in any therapeutic projects. As they put it, they “frankly did not see the point in trying.”

### Trajectory 2: I Gave It a Go, but Nothing Came of It — Being Met by a Therapist Representing a Rigid and Unhelpful System

Participants in Trajectory 2 also described negative preconceptions of coming to therapy. Some had difficulty with trust or did not see the need for treatment; others had heard negative rumors about mental health care or had friends with negative experiences. They were, however, willing to give it a chance, despite a less-than-optimal starting point:

No… I knew what I knew about the CAMHS, that they were no help, and that it was just crap and all that, but then I chose not to listen to those things […] So in a way I have really always thought that… maybe I can counterprove that in one way or another. (Participant 2).

The participants described feelings of apprehension and insecurity prior to the first contact. Some of the participants experienced the opportunity to bring a safe and trustworthy adult to the first meeting as a positive sign of flexibility in the system, thereby eliciting hope. Others experienced a problematic first meeting when their parents were invited to the first session without giving the adolescent an opportunity to comment on the invitation. Despite their varied experiences of entering the health care system, the participants in Trajectory 2 had sufficient hope to choose to continue to give treatment a chance—often for prolonged periods of time. Nevertheless, in the end, none of them felt their efforts had paid off. What, then, kept them going?

Unlike the participants in Trajectory 1, who experienced a rapid loss of hope, participants in Trajectory 2 experienced hope—either directly or vicariously—upon entering treatment. The three participants who had all their treatment experiences within Trajectory 2 experienced an OK first meeting with their therapists: “So it was really just going through things about why I was to be there and stuff, and that was fine, because they [the therapists] were, they were nice and all at the first meeting and stuff” (Participant 5). For two of these participants the experiences from the first session were reinforced by the vicarious hope of trusted adults:

Yes. And then I thought like people have said that [I should go to CAMHS] and I have sort of thought about it and now it was in a way like they respected it if I didn’t want to, and it was like, they, they, it didn’t say in my plan from the child protective agency that like I had to go to the CAMHS, it was like they said maybe that would be a good idea, and that they sort of really wanted me to do it and stuff, and I said like yes, fine, I can try it, like. (Participant 9).

Two other participants from Trajectory 2 had previous positive experiences from treatment that provided hope, and the last two participants had at least one significant other (i.e., a trusted school nurse, teacher, or parent), who communicated trust in treatment: “But then again the thing was that I saw my mum worrying, so I thought I have to do this [go see the CAMHS] if mum thinks it’s right” (Participant 2).

Despite this initial hope and continued willingness to give treatment a chance, all the participants in this trajectory reported that they and their therapists never managed to be on the same page, so to speak. This issue was partly related to experiencing a bad match or no chemistry with their therapist; their relational styles and preferences were simply not compatible: “I dunno, but it just didn’t work, the collaboration [with the therapist]. I don’t quite know how to explain it, it didn’t work. So I just held it all back and completely shut myself away” (Participant 4). For many participants, however, their experiences of not being able to open up or to communicate with their therapists in ways that made treatment helpful were related to the ways they were met by their therapists. Of the seven participants in this trajectory, five described how they experienced the therapists as disinterested in both getting to know them as people and exploring their problems: “Because there is something about sitting there and you are meant to tell someone something, and then they, it seems like they don’t care. That’s not very easy” (Participant 4).

The experience of therapists as seemingly disinterested as conveyed by their mechanical responses, such as repeating or responding without seeming to be engaged, reinforced the feeling that the therapists were “just doing their job.” Some of the participants also described how structural obstacles (e.g., notetaking, case notes, or assessments) could reinforce the notion that the therapists were merely doing a job and lacked interest in what the adolescent had to say, thus, making it even harder to open up.

And then… and also you have that thing that when you sit there talking to a psychologist, many of them tend to just sit there and write. And like, and then, it is better that they kind of pay attention to those who sit there and talk and show that actually you care what that person has to say. (Participant 5).

These experiences with a disinterested and disengaged therapist were compounded by the procedures of the mental health care system and the ways they influenced the therapists’ behaviors. For some participants, insecurity about information flow between the therapist and other adults (e.g., parents or child protection services) became a significant obstacle to engaging with the therapist: “It is hard to be honest then in a way because in a way you don’t know what is being done with the information you say, or, and you sort of don’t know what happens to it then” (Participant 9). The feeling that the therapists were in a rush and expected participants to start talking about their most difficult experiences right away made it harder for some participants to open up to them:

That the first appointments like, straightaway were about that, “so, you tried to kill yourself” and stuff like that, that straightaway you were onto the hardest, deepest darkest… that you never told anyone before. That you haven’t managed to say a word about before, you know. Mhm. And then you are supposed to start it with a total stranger. That is very hard. That it should be done a little more gradually. (Participant 1).

Most of the participants in Trajectory 2, thus, described the importance of balancing using time to get to know each other with courage to explore what is painful and show interest in what the adolescent experience as the problem. Several of the participants experienced that their therapists had their own agenda and seemed afraid to explore or misunderstood what was important treatment foci for the adolescents, thus increasing the feeling of not being seen:

But that wom… the CAMHS-woman, I don’t know what her education was called, but she never dared to, it seemed like she never dared to talk about the stuff that was actually important to me […] that, what I associated, associated with [CAMHS] like playing in that playroom, that was not then about talking about what might be important to me. We never talked about that. (Participant 4).

The fear of and the feeling of being misunderstood had a strong presence during treatment for many participants—for some, making it impossible to talk during sessions:

There was also one thing. Ahem… But then, it’s like, after all you get, you are very worried about being misunderstood when you are there. And things you say and stuff like that. Like that makes it very difficult to talk. But what happened was that when I actually managed to say something, it was sort of like I felt that it was misunderstood. And then I didn’t dare to say anything about that in a way. Yes, it’s like, when you say stuff then maybe it won’t be understood in the way you… you have to express yourself completely right, you know. And then it became like, yes, there, you’ve said it, so that’s how it will be, that is what is left standing. (Participant 1).

This quote also describes the active role of the adolescents in trying to make therapy work and the responsibility they felt to ensure the therapist could understand them and meet them in ways that would be helpful. Participants said they experienced and endured great discomfort (e.g., sitting in sessions in which they could not manage to say anything and in which the therapist responded to their silence with silence), attempting to build trust in the therapist over time. Because many participants felt such responsibility to make therapy work, they subsequently blamed themselves when they did not manage to open up or say anything during sessions. Some participants also tried to address the unhelpful behavior of the therapist in an attempt to improve treatment.

I said it several times like that, because she [the therapist] said like yes, or I said like I don’t feel it’s safe to talk to you or I feel that it’s uncomfortable, and then she said like yes, why is that, and I said that well, I don’t like you repeating everything I say in a way, the way you expect to get to know everything about me without me knowing anything about you, and I was just honest about it and stuff, eh, but it sort of didn’t get any better. It was just that, after I said that I didn’t like her repeating everything I said, repeating it, or what I’m saying, she repeated everything I had said about why I didn’t like talking to her and I just. OK, she didn’t listen to what I said, really. (Participant 9).

Participants who experienced disrespect from their therapists (e.g., blaming them for being victimized in bullying experiences or talking to them as if they were little kids who did not understand) felt they were responsible for making therapy successful and attempted to provide feedback without experiencing that the therapist changed behavior: “But I don’t think she (the therapist) took it (the feedback) very seriously, really. Because it was a bit like yes, I raised it to her during the appointment, and then it was back to square one… next session” (Participant 11).

Without the therapists knowing it, the adolescents shared how they could test the therapists’ responses to some of their difficulties.

Yes, because what many do, what I did too, it’s like, you start with the small stuff, and start talking about some things you are having a hard time with, and stuff. And then, if you get like a feeling that he [the therapist] can’t be bothered listening to me or that nor is there a point in talking in a way, then you withdraw again in a way… then it gets, it gets even harder to raise it again. (Participant 5).

This last example describes several core experiences in Trajectory 2. Despite efforts on both sides, the therapists and adolescents never managed to establish a sufficient number of points of contact to become visible to each other. Thus, they continued treatment seeing one another through muddy water. The adolescent’s position and experience of the situation did not become decisive of therapist’s choices; the therapist remained unaware of the adolescent’s continued efforts to check whether therapy could help them, including therapist tests. The adolescents were never included in the therapist’s inner circle of information and remained unable to acquire enough information to understand the therapist and the project. Consequently, they did not come to trust how the therapist understood them and their problems, where the therapist’s allegiance was, and whether the therapists really cared about them and their problems. Despite a lingering hope that therapy would help them, these experiences hindered a metamorphosis that could have made their efforts worthwhile. Importantly, the lack of therapist transparency could even alter the entire experience of treatment after the fact, as Participant 3′s experiences illustrate. Upon receiving her medical end report, she realized that her therapist had not been transparent about her diagnosis, amplifying the feeling that the therapist had not understood her at all:

I was given my discharge note from the CAMHS. A year late, almost. But that was OK, I guess. Uhm, but suddenly I found out that I have like four, five different diagnoses that my parents knew about but that I wasn’t told about. […] So, I lost in away very much my faith in the system again then. So, that’s why I can’t be bothered going into any new treatment even if I was to need it. […] Like, it was…it doesn’t seem like that doctor really understood what I meant and told her. Because what she writes is something completely different from what I meant. (Participant 3).

### Trajectory 3: Something Good Came of It — Being Met by a Therapist Who Cares and Wants to Help

The therapist was the main reason why something good came from the treatment contact for seven of the eight participants who shared their experiences in Trajectory 3. A clear improvement in symptoms in the last participant led to her finding the contact helpful and meaningful although she did not have an optimal match with her therapist and thought her improvement would have happened faster if the match had been a better one.

For the seven participants for whom the contact with the therapist was experienced as decisive for their therapy outcomes, the therapist represented a range of relational facilitators. Upon entering therapy, most of those participants had felt insecure and uncertain about what was expected from them, and therapy was a novel and scary avenue:

It [therapy] was, after all, completely new to me who was 11, so I was not used to talking to strangers. And then suddenly being sat with an unfamiliar person and being meant to talk completely openly about how you felt, it was… it becomes all too much in a way. (Participant 8).

The therapists seemed to face the task of transforming from an unsafe stranger into a safe, competent, and benevolent adult. The key to this transformation, in the eyes of the participants, was found in the combination of engagement, transparency, and warmth. This culminated in an experience of the therapist really wanting to help the adolescent: “So I felt some of that humanity then, that erm in [location 1] they wanted to help me because they saw that things were not as they should be” (Participant 2). The therapist’s effort to create a room in which the adolescent could get a glance of the therapist as a person and in which the therapist showed commitment in getting to know and understand the adolescent as a person rather than a client or a representative of a diagnosis was very important in this respect:

It was very much like the way she [the therapist] was, just the person she was too, the way she, her entire body language and the way she talked to me, especially about me, made me feel like she was genuinely interested in me in a way that, even if I sat there and was completely closed and cranky and (laughs) didn’t want to be there at all, right, I was super cranky, I just sat there and nearly cried and just… get it over with, but… but she got me in a way, it developed, right, to her, I saw that, in a way, there was something because she managed to… yes, because of the interest mainly, that she showed. (Participant 1).

The therapist’s continued engagement and interest in the adolescent’s perspective and experience of the situation and the willingness to see beyond problem behaviors was important for many participants: “I think it in a way has to do with how I was met, like, and that I was not given up on due to the cattiness and silence, like” (Participant 6). The therapist’s ability to cope with the adolescent’s silence was experienced as significant in this respect. Several participants had experienced that they didn’t manage to, dare to, or want to talk during sessions—most often because they did not feel they knew the therapist well enough, they were uncertain about how the therapist would understand what they said, or they were uncertain about how the information they shared would be used and who would access it. The way the therapists reacted to the adolescent’s silence was, therefore, often decisive for the participant’s experience of being in therapy and their faith in their therapist because the therapist’s reaction in this situation became a symbol of the therapist’s competence to handle difficult situations, the strength of the therapist–client relationship, and the therapist’s allegiance:

She [the therapist] sort of understood exactly what I needed, and she understood me in a way, so many times if I couldn’t manage to, she could in a way speak for me, erm, in a way that made that OK, because she understood me anyway. (Participant 7).

Many participants experienced great variation in their preferences and capacity for day-to-day activities, often in an unpredictable manner. The therapist’s sensitivity to variation in the adolescent’s capacity and preferences and the therapist’s willingness to be flexible and adjust the focus, expectations, and activities accordingly were very important for the participant’s experience of meeting a treatment system that could help them. Part of this flexibility also pertained to the clinic’s organization, including the therapist’s access to different treatment rooms and the freedom to engage in different activities to match the adolescent’s needs:

We [the therapist and I] could go out if, if I wanted to instead of like being in that room and focusing on me, like. So that was pretty lovely that you, yes, it didn’t turn out like I imagined. And but, yes, I chose to believe that it is, that I got her as a psychologist, that this determined it, that she could vary it that much. (Participant 6).

Over time, participants’ trust in their therapists increased. This was made possible through a combination of factors. In addition to aspects presented above, the opportunity to have the same therapist over time and the therapist’s ability to keep their word and show they were reliable was experienced as important. Finally, trust was facilitated by the therapists’ willingness to be transparent about their assessments, reactions, and thoughts, which were important prerequisites: “Because they [the therapists] explain too. They don’t just ask us to recount to them. They get impressions and then they say what they think” (Participant 12).

Meeting a therapist who really wanted to get to know and understand the participants and help them was the key factor contributing to participants being nudged into Trajectory 3. This made it possible to feel that therapy was worthwhile even in the face of quite a few obstacles, including misunderstandings and inattentiveness, insecurity regarding confidentiality, unsafe waiting rooms, and discomfort with the introduction of the parents’ perspective in therapy. However, when the therapist as a person became such a pivotal person, the match with the therapist and the opportunities for continuity became central to the continued benefits of treatment, thereby introducing a sense of vulnerability and fragility. Moreover, therapists were often on trial without knowing it; for example, an adolescent’s perspective on events, such as holidays and illnesses, could differ from that of the therapist. Any discontinuations in treatment, even short absences due to holidays or illnesses, could elicit old coping mechanisms and/or spark profound insecurity in the adolescents:

When you have gone for 4 weeks without [therapy], then I feel that, I have gone for 4 weeks without, why should I start up again? I have in a way managed, and then it sort of starts… when it was like regular, right, and we [the therapist and I] chatted lots and stuff I felt like in a way a bit of an effect of it, that here is someone who can talk. But then you start very much like, I can cope on my own, right. And because of that it’s a little bit harder to start now [after the holidays] because then, then is, then you get going again after those 4 weeks a bit and think that I can manage on my own. And then you start building that barrier again and stuff, like I can fix this myself, I don’t need you and stuff. […] So I feel that the first time after the holidays and after a break from the CAMHS then the first afterward like the first meeting that first time, right. That in a way you have to feel that chemistry and feel that feeling that… I think that either I will get there, or I will be very motivated and think that yes, it’s just stuff for me, I want to continue, or it will just confirm in a way what I already think about not needing it really. (Participant 1).

Participants in Trajectory 3 overcame these barriers together with the therapists, who used their attunement, sensitivity, and flexibility to safeguard the adolescents, convincing them of the therapist’s commitment to their common process:

Then it was sort of like, [I] am home with a sick child today, so I can’t make it. And that in a way made me feel a little more secure, because if someone from the CAMHS had made the call, I might have thought that oh, is it because she [the therapist] didn’t want to meet me today, like, that she, that she called in “sick” (laughs), and then she’s actually at the CAHMHS after all, but seeing some other patient she likes better, and starting to think like that. (Participant 6).

A closer look at Trajectory 3 points to important variation. One participant did not experience the therapist as the most important reason why treatment became worthwhile. Rather, she experienced differences with the therapist that indicated they were not a match. She experienced the therapist as somewhat unpredictable and unstable, felt the focus of treatment bounced from one thing to another, and often felt the therapist was in a rush, eliciting feelings of being a burden and unwelcome.

No, because you feel like you are under a great time pressure, that you in a way, you don’t feel completely wanted there, ever. When the first thing they [the therapists] say is hi, yes, sorry, I have an appointment at four, so you have to be done before that. […] Yes, it’s a bit like sorry for being here, I just need a little help. (Participant 8).

Nevertheless, she clearly experienced the treatment contact as meaningful. First, she experienced a clear improvement in symptoms and appreciated the tools she could use to cope with her symptoms, thus, enabling her to function better in her daily life. However, upon closer examination of the data, there were also significant relational facilitators, despite all the relational and structural obstacles. She experienced the unhurried time the therapist took to get to know her and the opportunities she was given to get to know the therapist in the beginning: “No, what they [the therapists] did then was that we took… erm, little by little, by making frequent appointments and allowing me to get to know them a little better” (Participant 8). Moreover, she experienced the feeling that the therapist wanted the best for her, took the time to explain mental health care thoroughly, emphasized confidentiality, and respected her boundaries:

P: But then we arranged (the therapists and I) it like I was to tell them if we got to, if I, if anything came up that I didn’t want to talk about, I was to tell them. …That we stop here, this stuff we will not talk about.

I: And they respected this?

P: Yes.

I: That’s really good, were you sort of the one telling them that you had to have rules like that, or did they suggest it?

P: Erm, they suggested it. And then they said that if there is, if we get to something you just need to tell us if you don’t want to talk about it or don’t reply. (Participant 8).

Finally, the participant had support outside treatment, which helped her to hang in there:

Like… yes but I had one, I discussed it often with my foster mother and said that I didn’t want to go there [CAHMS] due to the psychologist I was seeing. But then… but she said I had to just bite the bullet and that I got the psychologist I got. (Participant 8).

This last example is important to understand the nuances of how relational and structural facilitators and obstacles interact and create beneficial outcomes even in the face of substantial relational obstacles.

## Discussion

All the participants in this study entered therapies that were considered to have a difficult starting point in that they were initiated by adults. As we have seen, however, this shared starting point did not dictate the adolescents’ experiences of therapy. The evolving interactions between the adolescents and their therapists resulted in three distinct experiential trajectories through treatment. The findings show how therapy with a difficult starting point can fail from the perspective of the adolescent client. The findings also show the potential for helpful therapeutic processes, also when adolescents have come reluctantly at the initiative of others and even when the adolescents have prior negative therapy experiences. The presented findings, therefore, refine and widen our knowledge about what differentiates helpful from unhelpful clinical encounters from the perspective of adolescents who initially did not think they needed therapy.

Looking at the findings more in detail, we see that participants’ experience of the interactions with their therapists and the hope they developed became decisive for their journeys through mental health care. Our findings, thus, concur with previous research on alliance formation in adolescent therapy (Everall and Paulson, 2002; Martin et al., 2006; Binder et al., 2011; Gibson et al., 2016; Lavik et al., 2018; Løvgren et al., 2019) and expand the picture by showing how adolescents navigate both relational and structural facilitators and obstacles to get the most from therapy even when therapy is initiated by adults. Our findings also suggest that the alliance construct might have shortcomings in representing the breadth of relational experiences found in the adolescent client population. The real relationship is a construct that defines the need for genuineness and realism in treatment relationships (Gelso, 2011), and it has been found to be independently associated with outcomes in adult populations (Gelso, 2011). One hypothesis generated by our data and the relevant literature is that the construct of the real relationship (Gelso, 2011; Råbu and Moltu, 2020) developed alongside the alliance construct in adult psychotherapy but less applied and developed for the adolescent population could be beneficial and, therefore, should be researched in this context.

Trying to understand these findings, we see that the adolescents and their therapists have two particular difficulties to overcome in establishing a therapy that the adolescent experiences as helpful. First, an asymmetric client–therapist relationship is in tension with the developmental tasks of independence and autonomy for the adolescent, the latter making adolescents sensitive to any sign of a hierarchy in the relationship (Shirk et al., 2011). Second, therapeutic models that assume clients have entered therapy voluntarily do not typically match actual conditions in which adults have often initiated the therapy.

For the adolescent client coming reluctantly to therapy, the therapist is an unsafe stranger, a representative of the adult world, and consequently a generic figure met with expectations based on the adolescent’s prior experiences. The adolescent does not yet know what to expect and whether the therapist will be an ally in the adolescent’s life or a competitor in the areas of influence and agency. We can, therefore, understand why participants’ experiences of therapist transparency, benevolence, and authenticity would differentiate between trajectories. For participants in trajectory 3, their therapists develop from a generic representative of adulthood into an individual adult person with whom they could engage. Values of engagement, support, and acceptance were cultivated through behaviors enabling the participants to believe that the therapist was interested in their perspective. Transparency enabled participants to understand what the therapist did and why. Authenticity or genuineness helped participants get a sense of who the therapist was. In keeping with this, participants in all three trajectories emphasized the importance of allowing time in the beginning of therapy for adolescent clients and their therapists to get to know each other. This was experienced as particularly helpful when therapists were willing to let the adolescents get to know them a bit as persons and, conversely, to show interest in knowing the adolescents as persons. Participants subsequently referred negatively to therapists who seemed to “merely be doing a job.” This finding is in line with research showing that adolescents conceptualize positive therapeutic relationships being like adolescent–adult friendships (Everall and Paulson, 2002; Gibson et al., 2016; Løvgren et al., 2019).

The therapists’ tasks are not easy, though. Many young clients show their dissatisfaction by disengagement and silence rather than confrontation (Gibson and Cartwright, 2013). Moreover, therapists and adolescents perceive the developing alliance differently (van Benthem et al., 2020). As participants in this study share, many adolescent clients show their insecurity with challenging behavior, hide their true feelings, and secretly test therapists to see if they really care. In this study, child protective services were involved in the lives of seven of the participants and had initiated therapy for five of them. More than half of the participants in the study, therefore, had had severe negative relational experiences prior to therapy. Although analysis shows that these experiences did not determine which trajectory participants would follow, the participants acknowledged that their previous experiences did influence their experience of therapy.

Therapists, thus, often struggle to detect the adolescent’s voice. Clinical feedback systems have, therefore, been suggested to support therapists in their assessments (Bickman et al., 2011; Deighton et al., 2014; Gondek et al., 2016) and strengthen client collaboration (Solstad et al., 2019). Some such systems are tailored to young clients (Kelley and Bickman, 2009). Therapists also have to be aware of how their attitudes toward the adolescent client influence their clinical judgment (Strupp, 1993). Adolescence is an age of rapid change and is often associated with strong expressions of emotion and high levels of stress. Moreover, adolescent clients are often considered a difficult group to reach and treat (Everall and Paulson, 2002). Therapists, thus, risk discounting adolescent dissatisfaction in therapy as merely an expression of youthfulness or the nature of adolescents rather than an indicator of a specific and unique experience that should be given weight in guiding clinical practice. For the therapist too, then, the adolescent client has to transform from a generic representative of adolescence into an individual adolescent person if their therapeutic project is to succeed.

Despite these challenges, the presented findings point to a great potential in adolescent therapies initiated by adults. Although two participants experienced a rapid loss of hope upon entering mental health care and abandoned the idea of therapy within the first few sessions, most participants in this study continued in mental health care and remained open to the idea of therapy over time. Even participants in Trajectory 2, who experienced that their efforts did not pay off, utilized a lingering hope to repeatedly attempt to establish a therapeutic project with their therapist. This investment represents an opportunity for therapists and the health care system to reach this group and change practices to help them benefit from treatment. The difference between Trajectories 2 and 3 and between the five participants who switched trajectories when changing therapists or clinics suggests the vital role of successful therapist behaviors in eliciting hope in adolescent clients. On a more concrete level, participants shared how therapists’ behaviors that indicated individual tailoring based on the therapist’s precise perception of the adolescent’s needs and perspectives (e.g., personal texting while having a sick day) was important as it indicated that the therapist would go the extra mile. Although not taken for granted, these therapist behaviors were experienced as an essential adjustment to the adolescent’s specific needs to keep their relational insecurity from hampering the therapeutic relationship. This finding is consistent with previous research indicating that a strong working alliance is particularly important for adolescents with poor attachment experiences (Bucci et al., 2016). It also illustrates how sensitivity to the adolescents’ difficulties and life situations as well as to their experiences of therapeutic encounters allows tailoring of treatment to the individual.

We emphasize that adolescent clients experience agency in therapy and are active participants in creating therapeutic change rather than passive recipients of therapy (Gibson and Cartwright, 2013; Løvgren et al., 2019). Previous research shows how adolescent clients make active choices by staying in therapy or quitting (Block and Greeno, 2011), by deciding what to say and when (Løvgren et al., 2019). Our findings concur but nuance the picture by illustrating how adolescent clients can struggle with saying anything at all yet still be engaged in a therapeutic project, feeling great responsibility for the therapeutic process. Although the adolescent client may not exhibit overt action, they still manage agency by monitoring the usefulness and quality of the help offered and weighing their opportunities to influence the therapeutic process in a positive direction. That is, they try to find ways to put words to their experiences to break the all-consuming silence that characterizes therapy sessions in which the adolescent’s lack of words is met with silence on the part of the therapist. Many participants endured great discomfort that was elicited by the therapist’s behaviors and attempted to overcome the challenges they and their therapist faced (e.g., by giving the therapist feedback at a great cost). This clear display of agency and sense of responsibility for the treatment process is interesting, particularly given that all participants in our study entered mental health care at others’ instigation.

However, the findings reported in Trajectory 2 can also be seen as troubling and problematic in that the adolescents assumed responsibility for the quality of their treatment without correspondingly having access to power (Gibson and Cartwright, 2013). They, therefore, persevered in treatment over a prolonged period without experiencing benefit from it. These experiences depleted their faith in the system’s ability to help them. Therapists must, therefore, assess accurately how well treatment is meeting the adolescent’s needs and adjust it flexibly as needed. The organization of services (e.g., clinical procedures in the first session and assessment and diagnostic procedures) influences opportunities for the therapeutic flexibility needed to meet adolescent clients in ways that elicit hope and promote their willingness to invest in mental health care. Finally, the agency and responsibility assumed by the participants for the therapeutic process, developmentally significant as these are, must not be confused with the power and position to change the course of therapy; the main responsibility for the therapeutic process, we emphasize, still lies with the therapist.

## Limitations

In this study, we explored how adolescent clients who entered therapy at the initiative of others experienced therapy and what differentiated helpful from unhelpful therapy experiences from this starting point. The in-depth interviews with participants with firsthand experiences with adult-referred psychotherapy allowed for a nuanced and detailed exploration of what can contribute to positive therapy experiences even in the face of this obstacle. We note, though, that an adolescent can experience a therapist as disinterested while the therapist from her perspective feels very engaged and interested. Consequently, experiential data do not constitute the whole truth about an interpersonal situation. From a research perspective, participants’ experiences are nevertheless considered one truth in the sense that our experience and understanding of the world decides how we act in the situation relevant to the study. This study illuminates how therapy can be experienced from the perspective of adolescent clients although, for objective documentation of actual therapeutic interactions and their effectiveness, additional kinds of research may be called for. Because we were limited to the participants’ own reports on the type and amount of treatment they received, our interpretation of their subjective experiences may be missing some notable context.

Contextualizing the researchers, participants, and findings and being transparent about the research process are key to obtain good qualitative research. Moreover, there is no simple test to indicate the quality of the research. Rather, quality criteria, such as reflexivity, transparency, and contextualization, should be integrated into the research process and reflected in the way the research paper is written (see, e.g., Stige et al., 2009; Critical Appraisal Skills Programme, 2018), thus ensuring rigor and trustworthiness, the concepts corresponding to reliability and validity in quantitative research.

Finally, because 10 out of 12 participants were active in the user organization Forandringsfabrikken, their reflections on experiences in treatment clearly drew upon their engagement in efforts to improve services. Although the findings indicate that participation in the user organization was not based on mere negative experiences with mental health care, their involvement in advocacy is an important point to keep in mind when assessing the transferability of the findings.

## Conclusion

In this study, we investigated the experiences of 12 adolescents who had attended mental health care at the initiative of others. We found that the participants’ journeys through mental health care differed significantly as illustrated by three different trajectories. Some participants experienced a rapid loss of hope (Trajectory 1), others had lingering hope and stayed in treatment without experiencing any benefits (Trajectory 2), and the last group experienced some benefits from treatment (Trajectory 3). These findings point to the key role played by the therapist as a person and adolescents’ positive meetings with a safe, genuine, and flexible therapist who could make their treatment useful despite a less-than-optimal starting point. The results have important clinical implications, including the extent to which service organizations allow sufficient flexibility for therapists to ensure individualized treatment that meets the needs of adolescent clients. The findings also shed light on how adolescents are active participants in therapy, feel responsible for making the therapeutic relationship work, and often remain in therapy for a longer period without experiencing benefits even when they have entered therapy at the initiative of others. These findings also emphasize the importance of assessing adolescents’ experiences in therapy and adjusting treatment to facilitate helpful therapeutic processes.

## Data Availability Statement

The datasets generated for this study are not readily available because the data set is consisting of interview data, confidentiality can not be safeguarded. The data will therefore not be made available. Requests to access the datasets should be directed to SS, Signe.Stige@uib.no.

## Ethics Statement

The studies involving human participants were reviewed and approved by Regionale komiteer for medisinsk og helsefaglig forskningsetikk, Region Vest. Written informed consent to participate in this study was provided by the participants’ legal guardian/next of kin. Written informed consent was obtained from the individual(s), and minor(s)’ legal guardian/next of kin, for the publication of any potentially identifiable images or data included in this article.

## Author Contributions

SS is the project leader and initiated the project. She has been active in all phases of the project, including design, data collection, data analysis, and writing. TB has been active in all phases of the project, including design, data collection, data analysis, and writing. KL has been active in data analysis and writing. CM has been active in all phases of the project, including design, data collection, data analysis, and writing. All authors contributed to the article and approved the submitted version.

## Conflict of Interest

The authors declare that the research was conducted in the absence of any commercial or financial relationships that could be construed as a potential conflict of interest.
